# Immune response after vaccination of HIV infected individuals receiving HAART with overlapping gag peptides pulsed on autologous cells

**DOI:** 10.1186/1742-4690-9-S2-P115

**Published:** 2012-09-13

**Authors:** H Kløverpris, A Jackson, A Handley, P Hayes, J Gilmour, M Atkins, B Walker, J Ackland, M Sullivan, P Goulder

**Affiliations:** 1 University of Oxford, Oxford, UK; 2 St Stephens Clinic Chelsea and Westminister Hospital, London, UK; 3 Medicines Development Limited, Melbourne, Australia; 4 Imperial College London, IAVI Human Immunology Laboratory, London, UK; 5 Imperial College Healthcare, London, UK; 6 Ragon Insitute, Boston, USA; 7 GlobalBiosolutions, Craigieburn, Australia; 8 Oxford University, Paediatrics, Oxford, UK

## Background

HIV Gag specific CD4+ and CD8+ T cell responses are important for HIV immune control. Pulsing overlapping Gag peptides on autologous cells (Opal) has proven immunogenic and effective in reducing viral loads in multiple macaque studies, warranting clinical evaluation.

## Methods

We performed a phase I, single centre, placebo-controlled, double-blinded and dose-escalating study to evaluate the safety and preliminary immunogenicity of a novel vaccine approach Opal-HIV-Gag(c). This vaccine is constituted by 120 15mer peptides, overlapping by 11 amino acids spanning the entire HIV Gag C Durban consensus sequence proteome, pulsed on white blood cells enriched from whole blood using a closed system, followed by intravenously reinfusion. Patients with well controlled HIV on HAART received four vaccinations administered at week 0, 4, 8 and 12, and were followed up for 12 weeks post-treatment. Eighteen people were enrolled in three groups: 12mg (n=6), 24mg (n=6) or matching placebo (n=6). An additional group (48mg, n=2) was not evaluable. Immunogenicity was assessed by IFNγ ELIspot/ICS.

## Results

The constituent peptides in Opal-HIV-Gag(c) were antigenic in vitro using peptide stimulated PBMCs (median 30 fold increase). However, after vaccination with Opal-HIV-Gag(c), 1/6 at 12mg and 1/6 at 24mg had a 2 to 3 fold increase from Baseline of Gag specific CD8+ T cells at Week 14, compared to 0/6 placebo recipients. No Gag specific CD4+ T cell responses or overall change in Rev, Nef, Tat and CMV specific responses were detected. Marked, transient and self-limiting lymphopenia was observed immediately post-vaccination (4 hours) in Opal-HIV-Gag(c) but not placebo recipients, with median 1.72 to 0.67 million lymphocytes/mL for active groups (P<0.001), compared to 1.70 to 1.56 for placebo group (P=0.16).

## Conclusion

Despite the promising effect found in several Macaca nemestrina studies using this approach, Opal-HIV-Gag(c) was not significantly immunogenic in this population and improved methods of generating Gag-specific T-cell responses are required.

